# Supplementary material for: Unveiling the Bioactive Potential of the Invasive Jellyfish Phyllorhiza punctata Through Integrative Transcriptomic and Proteomic Analyses
Source: Biomolecules. 2025 Aug 4;15(8):1121. doi: 10.3390/biom15081121 (PMC12383608; doi:10.3390/biom15081121)
Supplement: Supplementary file 1 [file biomolecules-15-01121-s001.zip › Suplementary Material/FileS3/PEPFOLD4/XP_065055859_4972/clusters.html]

 PEP-FOLD clusters


Download archive of all models  here   

|  |  |  |
| --- | --- | --- |
| # | file | sOPEP |
| # cluster 1 - Size: 2 |  |  |
|  | XP\_065055859\_4972-model1 | -11.2065 |
|  | XP\_065055859\_4972-model1.1 | -9.29013 |
| # cluster 2 - Size: 1 |  |  |
|  | XP\_065055859\_4972-model2 | -9.30697 |
| # cluster 3 - Size: 10 |  |  |
|  | XP\_065055859\_4972-model3 | -7.25227 |
|  | XP\_065055859\_4972-model3.1 | -7.16669 |
|  | XP\_065055859\_4972-model3.2 | -7.16669 |
|  | XP\_065055859\_4972-model3.3 | -7.04513 |
|  | XP\_065055859\_4972-model3.4 | -6.90128 |
|  | XP\_065055859\_4972-model3.5 | -6.90128 |
|  | XP\_065055859\_4972-model3.6 | -6.69794 |
|  | XP\_065055859\_4972-model3.7 | -6.67905 |
|  | XP\_065055859\_4972-model3.8 | -6.29103 |
|  | XP\_065055859\_4972-model3.9 | -5.78121 |
| # cluster 4 - Size: 5 |  |  |
|  | XP\_065055859\_4972-model4 | -4.78855 |
|  | XP\_065055859\_4972-model4.1 | -4.731 |
|  | XP\_065055859\_4972-model4.2 | -4.16337 |
|  | XP\_065055859\_4972-model4.3 | -4.05302 |
|  | XP\_065055859\_4972-model4.4 | -3.90755 |
| # cluster 5 - Size: 10 |  |  |
|  | XP\_065055859\_4972-model5 | -4.12565 |
|  | XP\_065055859\_4972-model5.1 | -4.05416 |
|  | XP\_065055859\_4972-model5.2 | -3.80311 |
|  | XP\_065055859\_4972-model5.3 | -3.77735 |
|  | XP\_065055859\_4972-model5.4 | -3.72116 |
|  | XP\_065055859\_4972-model5.5 | -3.68481 |
|  | XP\_065055859\_4972-model5.6 | -3.67169 |
|  | XP\_065055859\_4972-model5.7 | -3.63435 |
|  | XP\_065055859\_4972-model5.8 | -3.55104 |
|  | XP\_065055859\_4972-model5.9 | -3.518 |
| # cluster 6 - Size: 11 |  |  |
|  | XP\_065055859\_4972-model6 | -4.10523 |
|  | XP\_065055859\_4972-model6.1 | -4.07702 |
|  | XP\_065055859\_4972-model6.2 | -3.99168 |
|  | XP\_065055859\_4972-model6.3 | -3.90475 |
|  | XP\_065055859\_4972-model6.4 | -3.81656 |
|  | XP\_065055859\_4972-model6.5 | -3.79672 |
|  | XP\_065055859\_4972-model6.6 | -3.67783 |
|  | XP\_065055859\_4972-model6.7 | -3.6445 |
|  | XP\_065055859\_4972-model6.8 | -3.56702 |
|  | XP\_065055859\_4972-model6.9 | -3.54797 |
|  | XP\_065055859\_4972-model6.10 | -3.50017 |
| # cluster 7 - Size: 8 |  |  |
|  | XP\_065055859\_4972-model7 | -4.0366 |
|  | XP\_065055859\_4972-model7.1 | -3.94851 |
|  | XP\_065055859\_4972-model7.2 | -3.76008 |
|  | XP\_065055859\_4972-model7.3 | -3.72679 |
|  | XP\_065055859\_4972-model7.4 | -3.66625 |
|  | XP\_065055859\_4972-model7.5 | -3.58967 |
|  | XP\_065055859\_4972-model7.6 | -3.58675 |
|  | XP\_065055859\_4972-model7.7 | -3.51647 |
| # cluster 8 - Size: 10 |  |  |
|  | XP\_065055859\_4972-model8 | -3.956 |
|  | XP\_065055859\_4972-model8.1 | -3.68236 |
|  | XP\_065055859\_4972-model8.2 | -3.66158 |
|  | XP\_065055859\_4972-model8.3 | -3.65236 |
|  | XP\_065055859\_4972-model8.4 | -3.63443 |
|  | XP\_065055859\_4972-model8.5 | -3.62938 |
|  | XP\_065055859\_4972-model8.6 | -3.57529 |
|  | XP\_065055859\_4972-model8.7 | -3.54957 |
|  | XP\_065055859\_4972-model8.8 | -3.54572 |
|  | XP\_065055859\_4972-model8.9 | -3.49074 |
| # cluster 9 - Size: 8 |  |  |
|  | XP\_065055859\_4972-model9 | -3.92118 |
|  | XP\_065055859\_4972-model9.1 | -3.66655 |
|  | XP\_065055859\_4972-model9.2 | -3.63095 |
|  | XP\_065055859\_4972-model9.3 | -3.57373 |
|  | XP\_065055859\_4972-model9.4 | -3.57043 |
|  | XP\_065055859\_4972-model9.5 | -3.5521 |
|  | XP\_065055859\_4972-model9.6 | -3.46822 |
|  | XP\_065055859\_4972-model9.7 | -3.4629 |
| # cluster 10 - Size: 8 |  |  |
|  | XP\_065055859\_4972-model10 | -3.78889 |
|  | XP\_065055859\_4972-model10.1 | -3.70821 |
|  | XP\_065055859\_4972-model10.2 | -3.68982 |
|  | XP\_065055859\_4972-model10.3 | -3.68482 |
|  | XP\_065055859\_4972-model10.4 | -3.60901 |
|  | XP\_065055859\_4972-model10.5 | -3.5056 |
|  | XP\_065055859\_4972-model10.6 | -3.47041 |
|  | XP\_065055859\_4972-model10.7 | -3.43461 |
| # cluster 11 - Size: 6 |  |  |
|  | XP\_065055859\_4972-model11 | -3.72874 |
|  | XP\_065055859\_4972-model11.1 | -3.65856 |
|  | XP\_065055859\_4972-model11.2 | -3.54983 |
|  | XP\_065055859\_4972-model11.3 | -3.46213 |
|  | XP\_065055859\_4972-model11.4 | -3.42747 |
|  | XP\_065055859\_4972-model11.5 | -3.34766 |
| # cluster 12 - Size: 8 |  |  |
|  | XP\_065055859\_4972-model12 | -3.64563 |
|  | XP\_065055859\_4972-model12.1 | -3.63979 |
|  | XP\_065055859\_4972-model12.2 | -3.60271 |
|  | XP\_065055859\_4972-model12.3 | -3.54584 |
|  | XP\_065055859\_4972-model12.4 | -3.54584 |
|  | XP\_065055859\_4972-model12.5 | -3.48251 |
|  | XP\_065055859\_4972-model12.6 | -3.45058 |
|  | XP\_065055859\_4972-model12.7 | -3.39623 |
| # cluster 13 - Size: 1 |  |  |
|  | XP\_065055859\_4972-model13 | -3.58458 |
| # cluster 14 - Size: 5 |  |  |
|  | XP\_065055859\_4972-model14 | -3.57841 |
|  | XP\_065055859\_4972-model14.1 | -3.54811 |
|  | XP\_065055859\_4972-model14.2 | -3.53774 |
|  | XP\_065055859\_4972-model14.3 | -3.50169 |
|  | XP\_065055859\_4972-model14.4 | -3.3764 |
| # cluster 15 - Size: 2 |  |  |
|  | XP\_065055859\_4972-model15 | -3.57336 |
|  | XP\_065055859\_4972-model15.1 | -3.45811 |
| # cluster 16 - Size: 2 |  |  |
|  | XP\_065055859\_4972-model16 | -3.57018 |
|  | XP\_065055859\_4972-model16.1 | -3.50898 |
| # cluster 17 - Size: 2 |  |  |
|  | XP\_065055859\_4972-model17 | -3.46318 |
|  | XP\_065055859\_4972-model17.1 | -3.43291 |
| # cluster 18 - Size: 1 |  |  |
|  | XP\_065055859\_4972-model18 | -3.46084 |
|  |  |  |
